# A Toxic Dose of Belladonna

**Published:** 1884-01-20

**Authors:** H. Gibbons


### A TOXIC DOSE OF BELLADONNA.

By H. GIBBONS, SR., M.D.

In the early part of September last I induced a patient who was suffering from pulmonary disease to go to Highland Springs, in Lake County, to the Sanitarium of Dr. C. M. Bates, formerly of San Francisco and Professor in the Medical Department of the State University. He improved somewhat, but complained of an annoying cough at night, and of night sweats. I directed for him a mixture containing tinc. digitalis, tinc. belladonnæ and sulph. morphiæ, a teaspoonful to be taken at bed-time. This would be about fifteen drops of the first, twenty of the second and one-tenth grain of morphia at a dose. He had been taking another preparation in tablespoonful doses, and this led him to the mistake of taking a tablespoonful of the new medicine. It follows that he swallowed at one dose sixty drops tinc. digitalis, eighty drops tinc. belladonna and nearly half a grain of morphia. The effect is so well described in a letter written by himself to his father that I will let him tell his own story:

"I took a tablespoonful on going to bed. The first sensation was a severe itching in my limbs like a million of fleas biting me. Then I dozed off and woke with a start to find my mouth as dry as parchment. I immediately got up to get a drink of water. I

stood on the floor and my legs shook as if I had the palsy. I finally tottered along and got a drink and came back to the bed and lay across it with my feet on the floor. By that time my mouth was all dried up again, and so I got another drink and then back to bed. I kept repeating this three or four times more, and then I wanted to throw up and could no longer stand. Getting down on my hands and knees, I threw up the water and some of the medicine, By this time the housekeeper was aroused. Knocking at my door she inquired if she could do anything for me. I could not speak. Then she asked if she should call the doctor. Again she asked the same question, and then I managed to say yes. I was still lying across the bed when the doctor came and put me to bed, when I became quiet.

"All this time my mouth was parched. At eleven o'clock the doctor left me, leaving two glasses of water on a chair near my bed. I would take a sip of water, doze off, awake with a dry mouth, take another sip and then doze off again. I kept this up until one glass had been emptied. I took up the other glass and held it in my hand to sip it as I had done the other. I did this for a while and then dropped it, breaking the glass and arousing the landlady again. She then called the doctor. I sleep in a double bed. The doctor came and slept with me the rest of the night, lying on the inside. I soon grew delirious and raved about setting type [he is a printer], distributing type, and talking to all the men up here. Then the doctor put a piece of wet flannel on my forehead I raved for quite a while, and finally could not speak, only whisper, and still I kept on raving. At last I could not even whisper and almost ceased to breathe. For two hours I remained in that way, the doctor not knowing whether I would live or die. But he stuck to me, keeping the wet flannel on my forehead and bathing and rubbing my legs. During these two hours my heart was beating like a hammer, and he could hear it while bathing my feet. Then I began to revive and commenced my raving again. I looked out the window and saw the biggest fire I ever saw in my life. The whole country was on fire and I wanted to get up and see it, but the doctor would not let me. Toward morning I began to regain my senses, and at six o'clock I got up. I then walked around until breakfast time and went in to take my breakfast. I sat down and called for a beefsteak. I took some butter on my knife to put on my bread. I glanced at the butter before spreading it, and it was covered with millions of little quicksilver balls. Everything I tried to eat was covered with quicksilver, and so I left the table. About an hour after the landlady persuaded me to go and try to eat again, and so I went in and took a glass of milk and a biscuit. Towards evening I was all right again, except my eyes. You may thank God and Dr. Bates that you did not have to come for my dead body. I feel better now than since I came here. The pupils of my eyes are about twice their natural size."

That a person under the influence of a toxic dose, the action of which is expended principally on the brain, should be capable of giving a statement of his own case so precise and intelligent, appears to me a remarkable circumstance. The predominant symptoms were those occasioned by the belladonna, though there can be no question that each of the three drugs modified the united effect in some degree.—*Pacific Med. Jour.*

